# Supplementary material for: Migration arrest and transendothelial trafficking of human pathogenic-like Th17 cells are mediated by differentially positioned chemokines
Source: Nat Commun. 2025 Feb 26;16:1978. doi: 10.1038/s41467-025-57002-6 (PMC11861662; doi:10.1038/s41467-025-57002-6)
Supplement: Supplementary file 1 — Supplementary Information [file 41467_2025_57002_MOESM1_ESM.pdf]

Migration arrest and transendothelial trafficking of human pathogenic-like Th17 cells are mediated by differentially positioned chemokines

Farhat Parween[1], Satya P. Singh[1§], Nausheen Kathuria[1§], Hongwei H Zhang[1§], Shinji Ashida[2§], Francisco A. Otaizo-Carrasquero[3], Amirhossein Shamsaddini[3], Paul J. Gardina[3], Sundar Ganesan[4], Juraj Kabat[4], Hernan A. Lorenzi[5], Deanna J. Riley[6], Timothy G. Myers[3], Stefania Pittaluga[6], Bibiana Bielekova[2], and Joshua M. Farber[1*]

[1]Inflammation Biology Section, Laboratory of Molecular Immunology, National Institute of Allergy and Infectious Diseases, National Institutes of Health, Bethesda MD 20892, USA

[2]Neuroimmunological Diseases Section, Laboratory of Clinical Immunology and Microbiology, National Institute of Allergy and Infectious Diseases, National Institutes of Health, Bethesda MD 20892, USA

[3]Genomic Technologies Section, Research Technologies Branch, National Institute of Allergy and Infectious Diseases, National Institutes of Health, Bethesda MD 20892, USA

[4]Biological Imaging Section, Research Technologies Branch, National Institute of Allergy and Infectious Diseases, National Institutes of Health, Bethesda MD 20892, USA

[5]Bioinformatics and Computational Biosciences Branch, National Institute of Allergy and Infectious Diseases, National Institutes of Health, Bethesda MD 20892, USA

[6]Laboratory of Pathology, Center for Cancer Research, National Cancer Institute, National Institutes of Health, Bethesda MD 20892, USA

[§]Authors contributing equally

*Correspondence: jfarber@niaid.nih.gov

Supplementary Figures 1-9

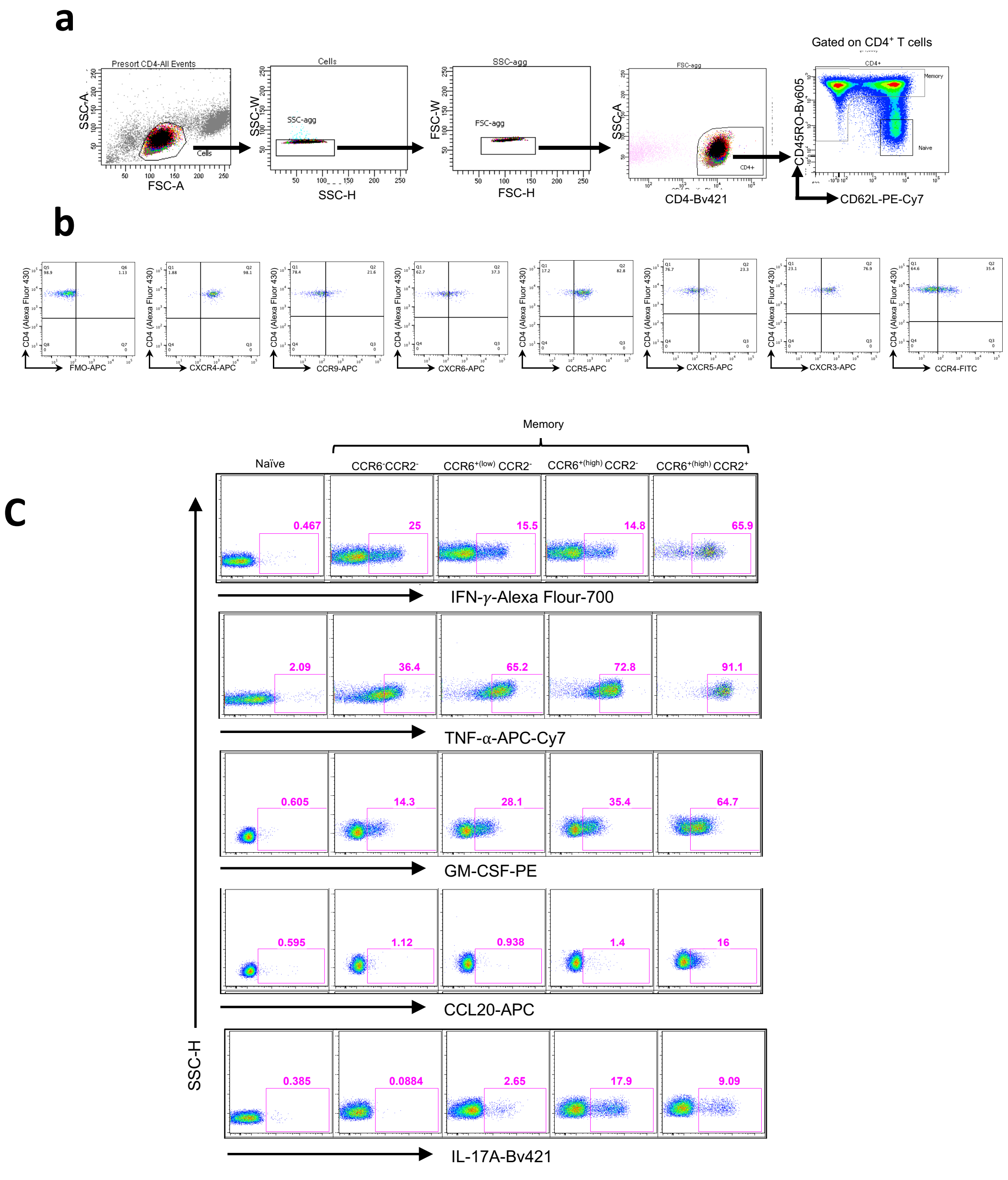

Supplementary Figure 1. Gating strategies. **a** Gating strategy to identify human CD4+ T cells for purifying cell subgroups as shown in Fig. 1a. This strategy was used for all experiments using purified subgroups of CD4+ T cells. **b** Gating strategy used to determine percentages of cells staining positive for chemokine receptors as shown in Supplementary Fig. 2b. **c** Gating strategy to determine percentages of cells staining positive for intracellular cytokines in CD4+ T cell subgroups as shown in Fig 1e.

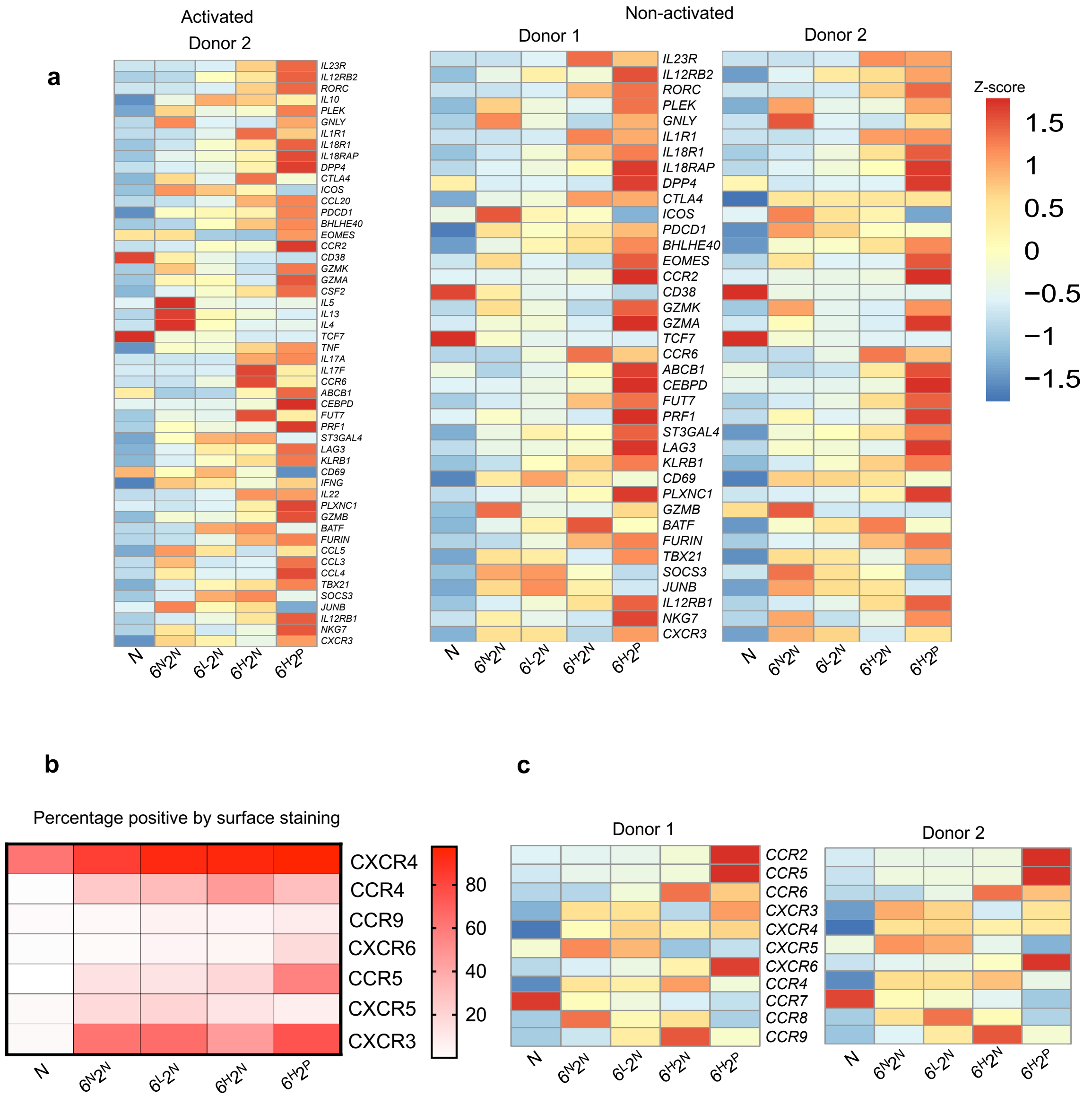

Supplementary Figure 2. Gene and chemokine receptor expression on the $CD4^+$ T cell subgroups. Human $CD4^+$ T cell subgroups were isolated by FACS from the blood of healthy donors as in Supplementary Fig. 1a and Fig. 1a. **a** Heatmaps from bulk RNA-seq showing the expression levels of genes (rows) associated with the pathogenic type 17 phenotype in activated cells from donor 2 and from non-activated cells from donors 1 and 2 in the purified $CD4^+$ T cell subgroups as indicated. Cytokine genes are not displayed in the heatmap from the non-activated samples. Color coding reflects standardized gene expression values (z-scores). **b** Heatmap showing averages of percentages of cells in the naïve and four memory subsets that were positive by surface staining for the indicated chemokine receptors from three donors. Source data are provided in the Source Data file. **c** Heatmaps showing the expression levels of chemokine receptor mRNAs in cell subgroups obtained from bulk RNA-seq from non-activated samples from donors 1 and 2.

Supplementary Figure 2

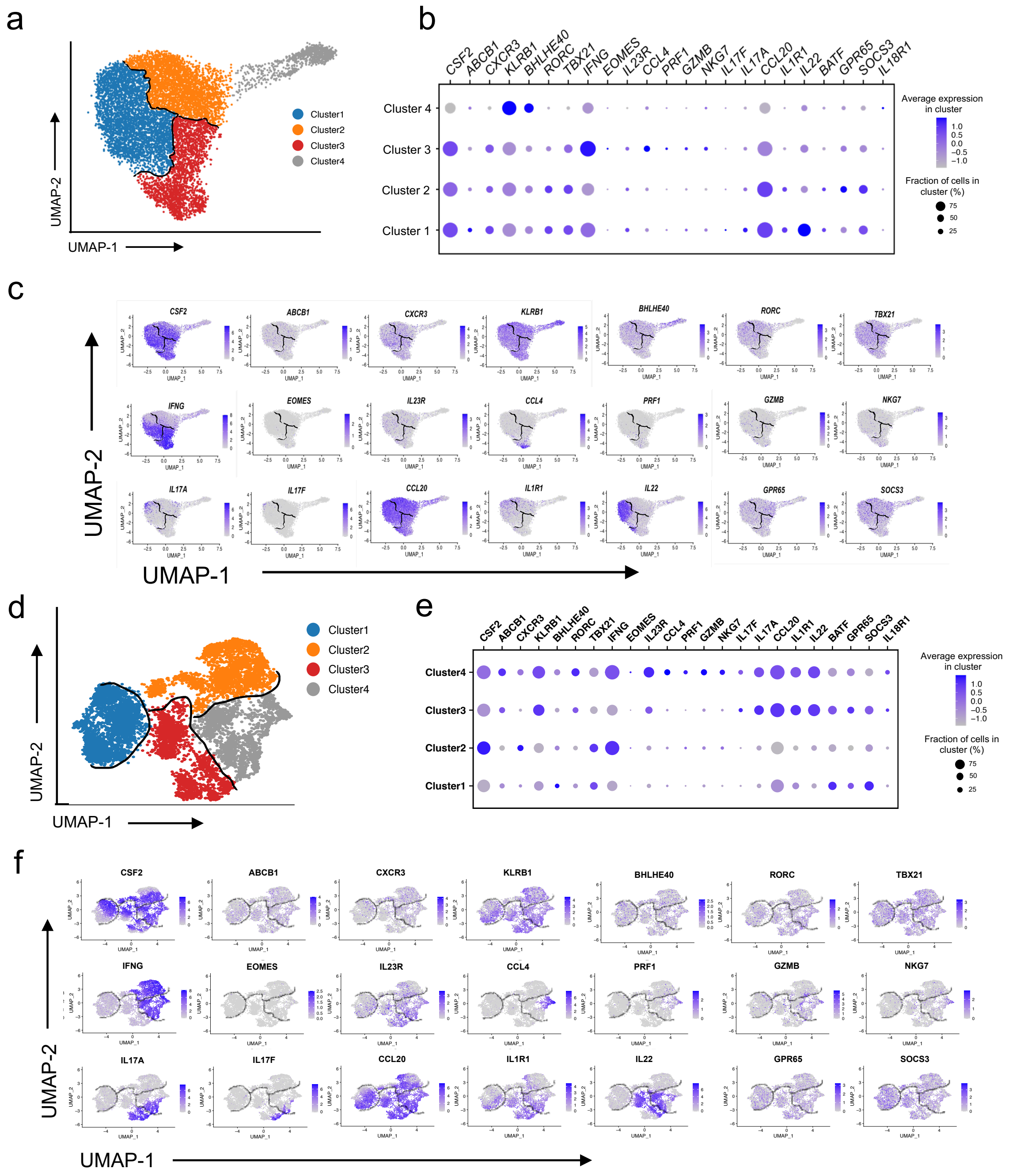

Supplementary Figure 3. CD4^{+}CCR6$^{+(high)}$CCR2^{+} T cells separate into clusters expressing *IL17A/F/IL22* vs. *IFNG*. Human CD4^{+}CCR6$^{+(high)}$CCR2^{+} T cells were isolated from the blood of healthy donors by FACS as in Supplementary Fig. 1a and Fig. 1a. **a** and **d** UMAP and clustering from scRNA-seq profiles of 8,957 CCR6$^{+(high)}$CCR2^{+} cells from Donor 2 (**a**) and 8,218 CCR6$^{+(high)}$CCR2^{+} cells from Donor 3 (**d**) where each dot represents a single cell, with color codes of clusters defining cells with similar transcriptional profiles. **b** and **e** Dot plots displaying gene expression for genes of interest from donor 2 (**b**) and donor 3 in (**e**), where the size of a dot indicates the percentage of cells in a cluster expressing the indicated gene and the shading represents the average expression level of the gene in the clusters' cells. **c** and **f** Feature plots of selected genes in cells in the UMAP scatter plots from donor 2 (**c**) and donor 3 (**f**), where the shading represents expression levels. Cluster borders are marked in black.

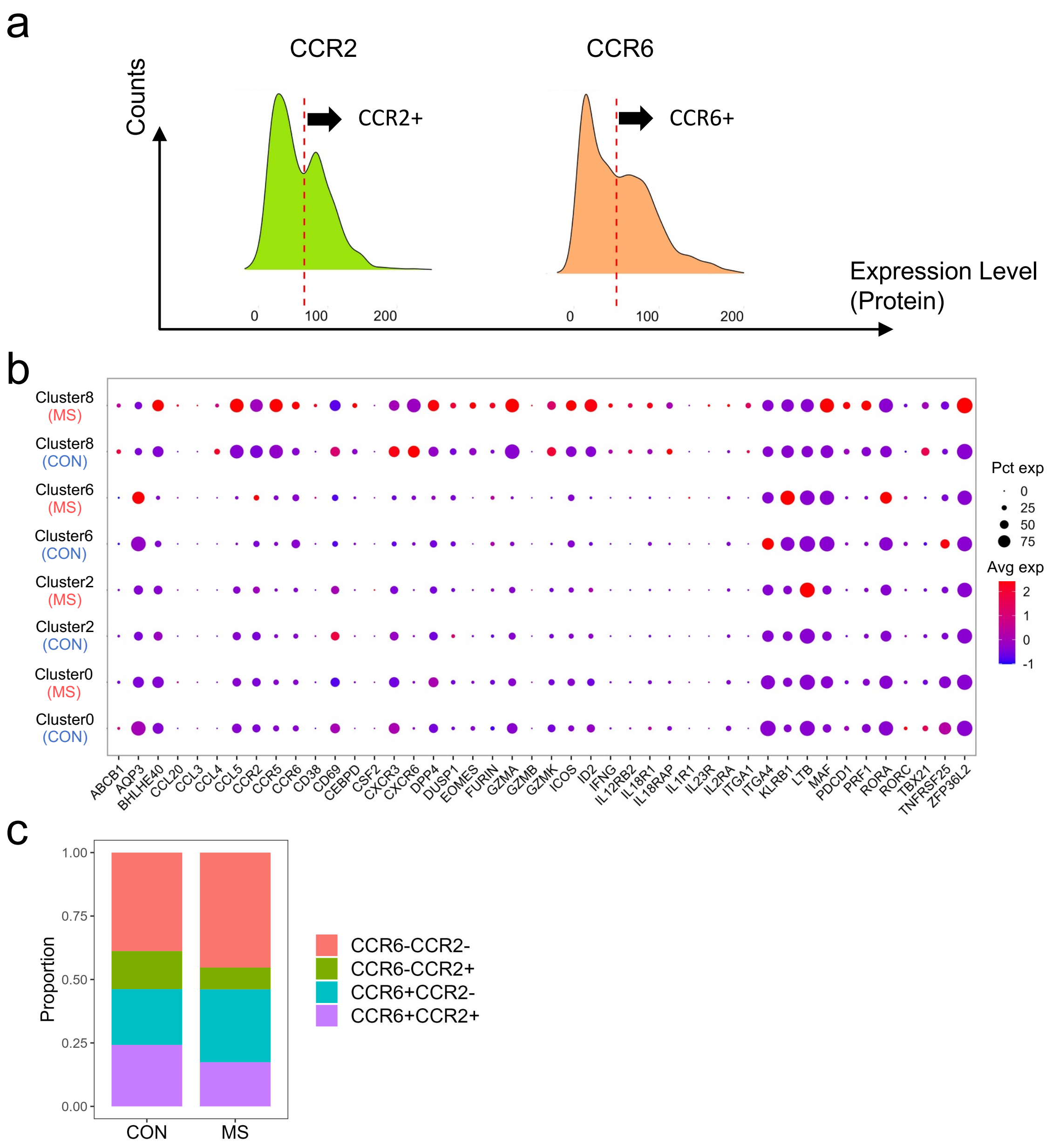

Supplementary Figure 4. CITE-seq analysis shows differences in UMAP cluster 8 in expression of pathogenicity-associated genes in CSF cells from MS versus control participants. **a** Identification of $CCR2^+$, $CCR2^-$, $CCR6^+$, and $CCR6^-$ cells using antibody-derived data from CITE-seq from Batch 2. **b** Dot plot visualizing the expression of key pathogenic type 17-associated genes for the cells from MS versus control (CON) participants for UMAP clusters 0, 2, 6, and 8 (see Fig. 3a). The size of a dot indicates the percentage of cells in a cluster expressing the indicated gene and the shading represents the average expression level of the gene in the clusters' cells. **c** Proportions of cells from control (CON) and MS subjects analyzed according to surface expression of CCR6 and CCR2, using antibody-derived data from Batch 2. Source data are provided in the Source Data file.

Supplementary Figure 4

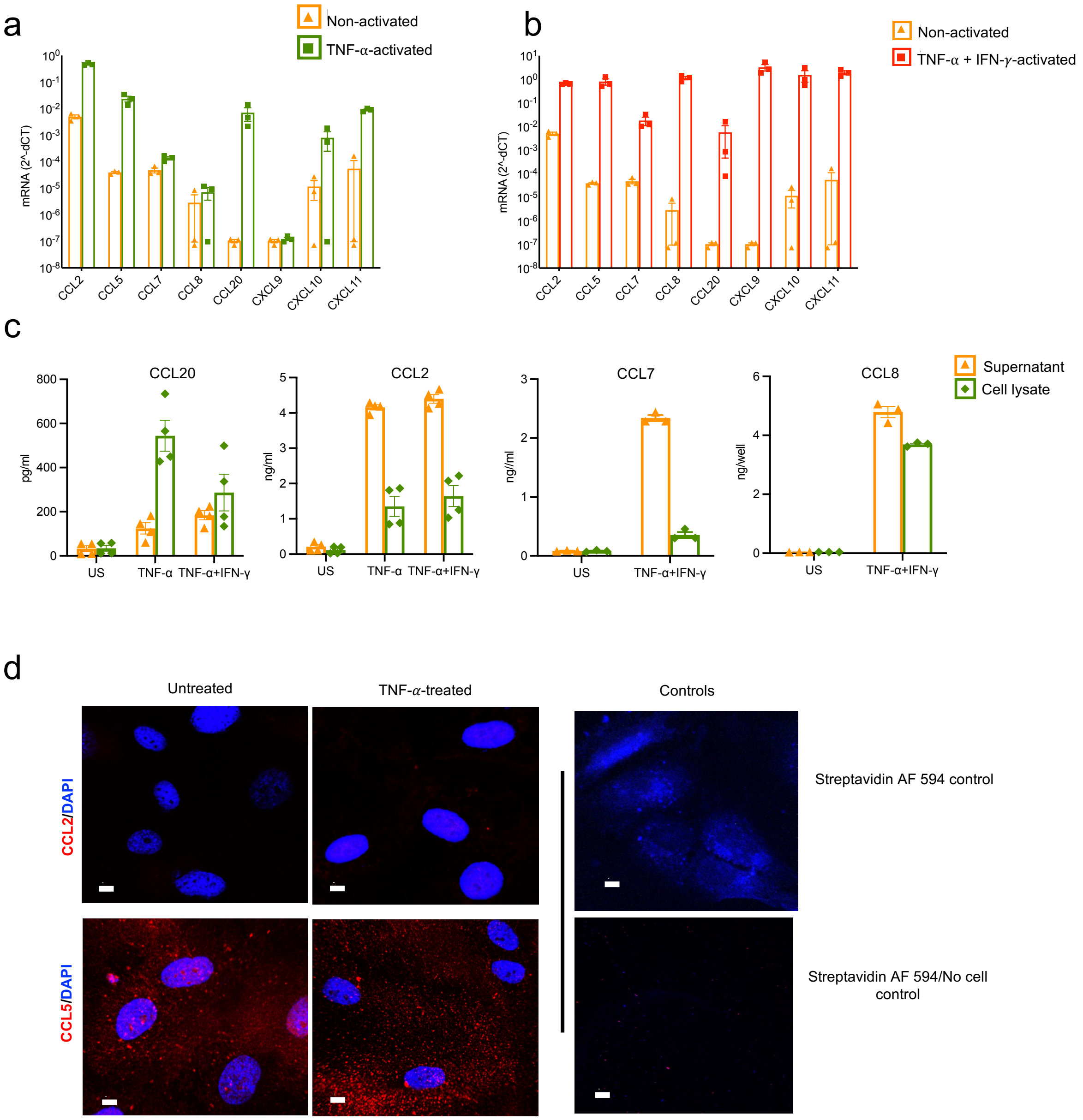

Supplementary Figure 5. CCR2 ligands produced by activated HUVECs are less cell-associated. **a** and **b** Real-time RT-PCR analysis of mRNA levels of chemokine genes in TNF-α or TNF-α + IFN-γ-treated HUVECs. Each symbol shows data from one of the three experiments shown. **c** Culture supernatants collected from the TNF-α or TNF-α + IFN-γ-treated HUVECs were assayed for CCL20, CCL2, CCL7 and CCL8 by ELISA. Each symbol shows data from one of the $\geq$ 3 experiments shown. **d** Confocal microscopy images at x 40 magnification of non-permeabilized, untreated or TNF-α-treated HUVECs after incubating with synthesized, biotinylated CCL2 or CCL5 and staining with DAPI for nuclei (blue). Chemokines were visualized using Alexa Fluor 594-streptavidin (red). The scale bars indicate 5 μm. Images are representative of at least three experiments. Control cells not incubated with chemokine and a fibronectin-coated slide without cells were treated with streptavidin-Alexa Fluor 594. Bars indicate means +/- SEM. Source data are provided in the Source Data file.

Supplementary Figure 5

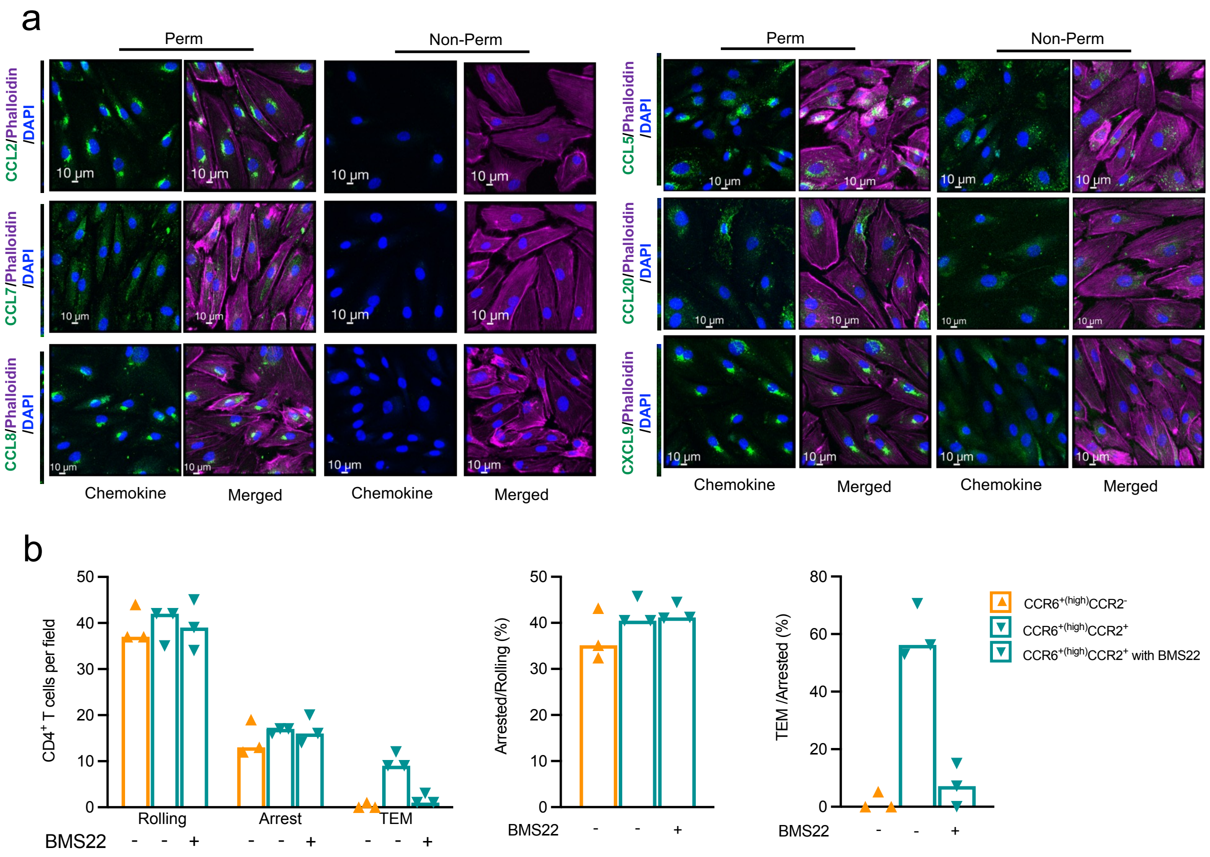

Supplementary Figure 6. CCR6$^{+(high)}$CCR2^{+} CD4^{+} T cells undergo transendothelial migration on HDMECs. **a** Confocal microscopy images at × 40 magnification of permeabilized and non-permeabilized TNF-α + IFN-γ-stimulated HDMECs immunostained for CCL2, CCL7, CCL8, CCL5, CCL20, and CXCL9 (green), and stained using phalloidin for polymerized actin (magenta) and DAPI for nuclei (blue). The scale bars indicate 10 µm. Images are representative of three experiments. Images from another of these three experiments are shown in Supplementary Figure 7. **b** Human CD4^{+} T cell subgroups were isolated by FACS from the blood of healthy donors as in Supplementary Fig. 1a and Fig. 1a. In the left panel are shown numbers of cells rolling, arrested and transmigrated on TNF-*α* + IFN-*γ*-activated HDMECs. T cells were either untreated (marked with a minus sign) or treated with the CCR2 antagonist, BMS22. Middle panel shows arrested cells as percentages of cells rolling and right panel shows transmigrated cells as percentages of cells arresting. Each symbol shows data from one of n=3 individual donors, analyzed from three separate experiments, and bars indicate medians. Source data are provided in the Source Data file and representative videos used to quantify rolling, arrest and transendothelial migration are provided in Supplementary Movies 28-30.

Supplementary Figure 6

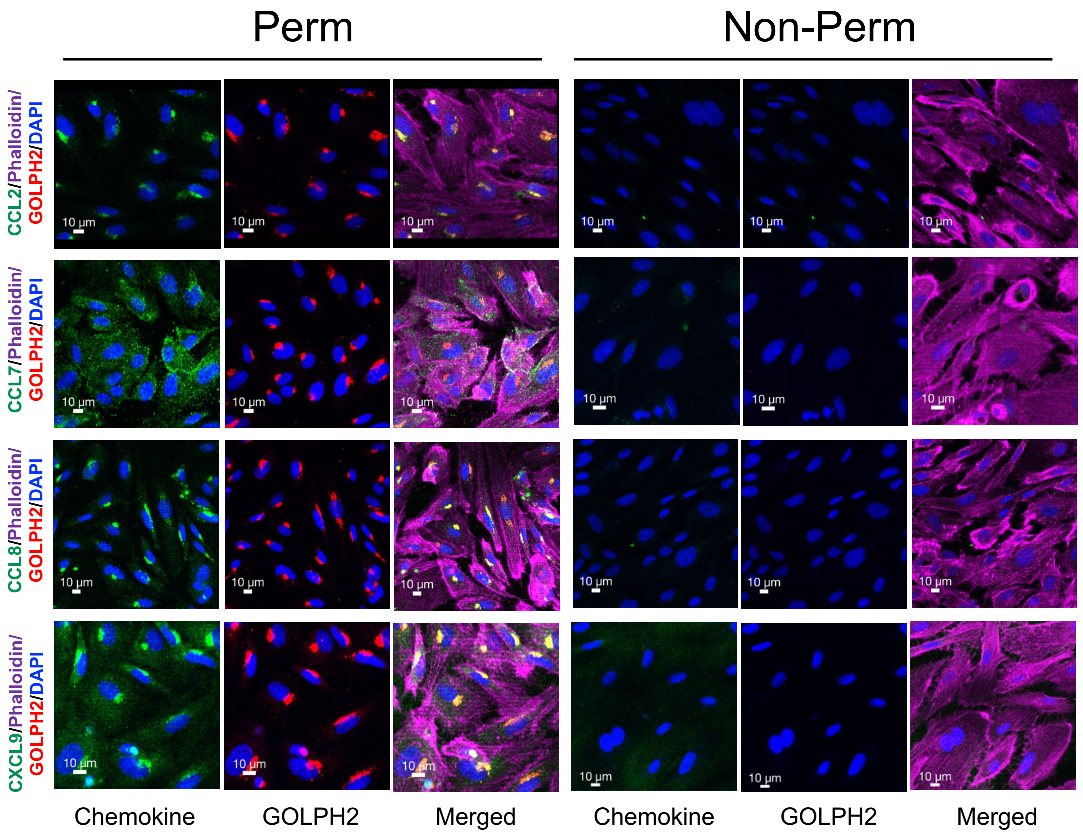

Supplementary Figure 7. Co-staining of chemokines and Golgi on HDMECs. Confocal microscopy images at × 40 magnification of permeabilized and non-permeabilized TNF-α + IFN-γ-stimulated HDMECs immunostained for CCL2, CCL7, CCL8, and CXCL9 (green), and stained using phalloidin for polymerized actin (magenta), GOLPH2 for Golgi (red), and DAPI for nuclei (blue). The scale bars indicate 10 µm. Images are representative of three experiments with HDMECs and three experiments with HUVECs. Images from another of the three experiments using HDMECs are shown in Supplementary Figure 6.

a

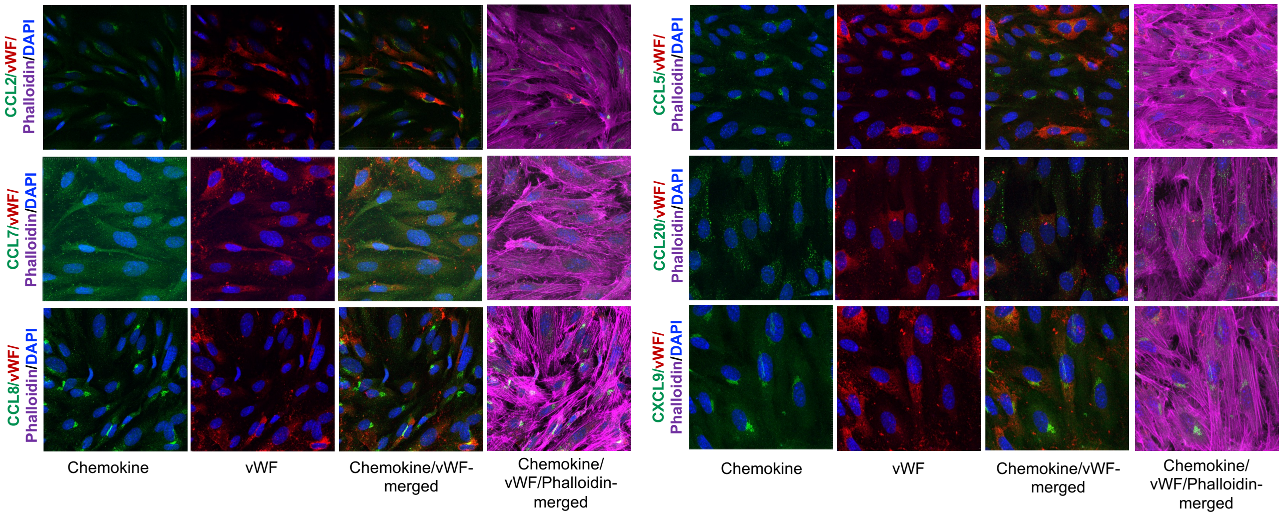

b

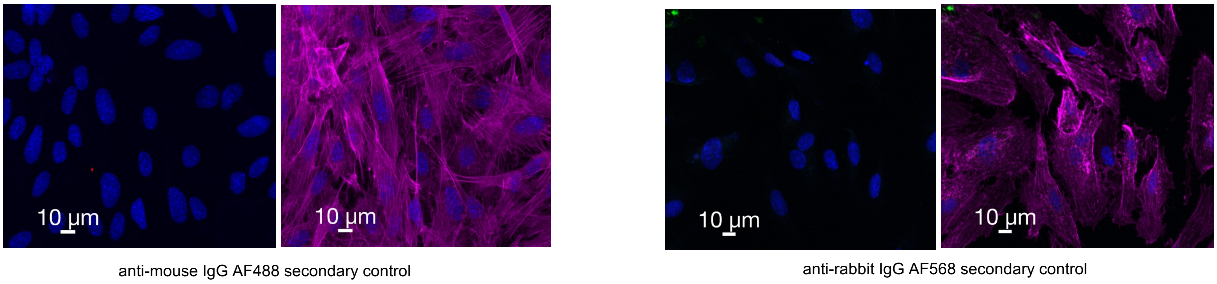

Supplementary Figure 8. Co-staining of chemokines and von Willebrand factor (vWF) on HUVECs. **a** Confocal microscopy images at × 40 magnification of permeabilized TNF-α + IFN-γ-stimulated HUVECs immunostained for CCL2, CCL7, CCL8, CCL5, CCL20, and CXCL9 (green) and for vWF to identify Weibel-Palade bodies (red), and stained using phalloidin for polymerized actin (magenta), and DAPI for nuclei (blue). Images are representative of two experiments. **b** Shown are secondary antibody controls for the staining for chemokines (anti-mouse IgG AF488) and GOLPH2 (Supplementary Fig. 7), and vWF (anti-rabbit IgG AF568), along with staining with phalloidin for polymerized actin (magenta) and DAPI for nuclei (blue). Images are representative of three experiments.

Supplementary Figure 8

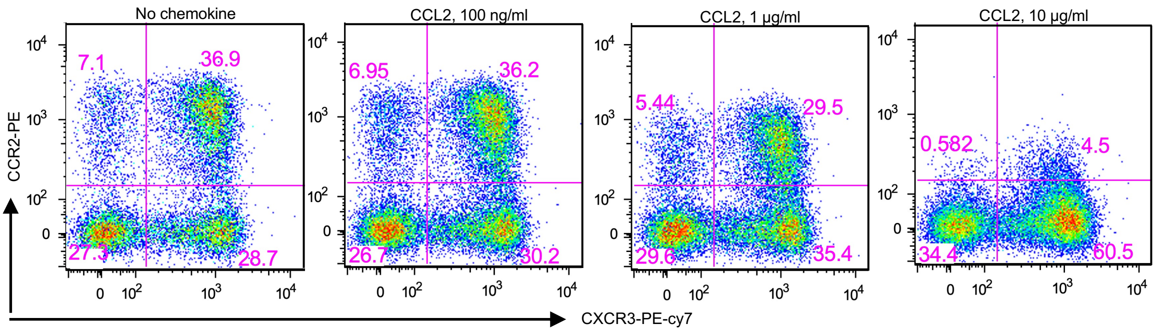

Supplementary Figure 9. CCR2 is not internalized on CD4+ T cells by 100 ng/ml CCL2. Human peripheral blood mononuclear cells from a healthy donor were treated with the indicated concentrations of CCL2 for 30 min at 37 °C and dot plots show staining for CCR2 and CXCR3 on CD4+CD45R0+ T cells, identified as in Fig. 1a. Results are representative of three experiments.

Supplementary Figure 9

Supplementary Table 1

Antibody	Source	Cat #	Dilution
anti–human CD4-Bv 421	Biolegend	317434	2 μL/10^6 cells
anti–human CD-45RO-Bv605	Biolegend	304238	2 μL/10^6 cells
anti–human CD62L-PECy5	Biolegend	304808	1 μL/10^6 cells
anti–human CD25-APC-Cy7	Biolegend	302614	2 μL/10^6 cells
anti–human HLA-DR-APC-Cy7	Biolegend	307618	2 μL/10^6 cells
anti–human CD127-FITC	Biolegend	351414	2 μL/10^6 cells
streptavidin-PE to use with anti-CCR2	Biolegend	405245	0.5 μL/10^7 cells
anti-human-IFN-γ	Biolegend	502530	5 μl/test in 100 μL
anti-human-IL-17A	Biolegend	512305	5 μl/test in 100 μL
anti-human-GM-CSF	Biolegend	502305	5 μl/test in 100 μL
anti-human-TNF-α	Biolegend	502944	5 μl/test in 100 μL
anti-human CCR2-PE	Biolegend	357206	2 μL/10^7 cells
anti-human-CCR6-PECy7	BD Pharminogen	560620	2.5 μl/10^6 cells
Biotinylated anti-human-CCR2	R&D Systems	FAB151B	10 μl/10^6 cells
anti-human-CCL20	R&D Systems	C360A	5 μl/test in 100 μL
anti-human-CCL2	R&D Systems	MAB679	1:100 dilution
anti-human-CCL5	R&D Systems	MAB278	1:100 dilution
anti-human-CCL7	R&D Systems	MAB282	1:100 dilution
anti-human-CCL8	R&D Systems	MAB281	1:100 dilution
anti-human-CCL20	R&D Systems	AF360	1:100 dilution
anti-human-CXCL9	R&D Systems	MAB392	1:100 dilution
anti-human-vWF	abcam	ab6994	1:100
anti-human-GOLPH2	Invitrogen	PA5-30622	1:100 dilution
anti-mouse-Alexa Fluor 488	Invitrogen	A-11017	1:500 dilution
anti-rabbit-Alexa Fluor 568	Invitrogen	A-11036	1:500 dilution
Phalloidin	Invitrogen	A-30107	1:400 dilution
DAPI	Invitrogen	62248	1 μg/ml
