# Supplementary material for: Migration arrest and transendothelial trafficking of human pathogenic-like Th17 cells are mediated by differentially positioned chemokines
Source: Nat Commun. 2025 Feb 26;16:1978. doi: 10.1038/s41467-025-57002-6 (PMC11861662; doi:10.1038/s41467-025-57002-6)
Supplement: Supplementary file 2 — Description of Additional Supplementary Files [file 41467_2025_57002_MOESM2_ESM.pdf]

Description of Additional Supplementary Files

Supplementary Movie 1. Videomicroscopy of naïve $CD4^+$ T cells from a representative experiment/donor for data in Figure 4a.

Supplementary Movie 2. Videomicroscopy of $CCR6^-CCR2^-CD4^+$ T cells from a representative experiment/donor for data in Figure 4a.

Supplementary Movie 3. Videomicroscopy of $CCR6^{+(low)}CCR2^-$ $CD4^+$ T cells from a representative experiment/donor for data in Figure 4a.

Supplementary Movie 4. Videomicroscopy of $CCR6^{+(high)}CCR2^-$ $CD4^+$ T cells from a representative experiment/donor for data in Figure 4a.

Supplementary Movie 5. Videomicroscopy of $CCR6^{+(high)}CCR2^+$ $CD4^+$ T cells from a representative experiment/donor for data in Figure 4a.

Supplementary Movie 6. Videomicroscopy of $CCR6^-CCR2^-$ $CD4^+$ T cells from a representative experiment/donor for pertussis toxin control data in Figure 4b.

Supplementary Movie 7. Videomicroscopy of $CCR6^-CCR2^-$ $CD4^+$ T cells from a representative experiment/donor for pertussis toxin treatment data in Figure 4b.

Supplementary Movie 8. Videomicroscopy of $CCR6^{+(high)}CCR2^+$ $CD4^+$ T cells from a representative experiment/donor for pertussis toxin control data in Figure 4b.

Supplementary Movie 9. Videomicroscopy of $CCR6^{+(high)}CCR2^+$ $CD4^+$ T cells from a representative experiment/donor for pertussis toxin treatment data in Figure 4b.

Supplementary Movie 10. Videomicroscopy of $CCR6^{+(high)}CCR2^+$ $CD4^+$ T cells from a representative experiment/donor for anti-CCL20 control data in Figure 4c.

Supplementary Movie 11. Videomicroscopy of $CCR6^{+(high)}CCR2^+$ $CD4^+$ T cells from a representative experiment/donor for anti-CCL20 treatment data in Figure 4c.

Supplementary Movie 12. Videomicroscopy of $CCR6^{+(high)}CCR2^+$ $CD4^+$ T cells from a representative experiment/donor for maraviroc control data in Figure 4d.

Supplementary Movie 13. Videomicroscopy of $CCR6^{+(high)}CCR2^+$ $CD4^+$ T cells from a representative experiment/donor for maraviroc treatment data in Figure 4d.

Supplementary Movie 14. Videomicroscopy of $CCR6^{+(high)}CCR2^+$ $CD4^+$ T cells from a representative experiment/donor for BMS22 control data in Figure 4e.

Supplementary Movie 15. Videomicroscopy of $CCR6^{+(high)}CCR2^+$ $CD4^+$ T cells from a representative experiment/donor for BMS22 treatment data in Figure 4e.

Supplementary Movie 16. Videomicroscopy of $CCR6^{+(high)}CCR2^+$ $CD4^+$ T cells from a representative experiment/donor for TNF-a treatment data in Figure 5a.

Supplementary Movie 17. Videomicroscopy of $CCR6^-CCR2^-$ $CD4^+$ T cells from a representative experiment/donor for TNF-a plus IFN-g treatment data in Figure 5a.

Supplementary Movie 18. Videomicroscopy of $CCR6^{+(low)}CCR2^{-}$ $CD4^{+}$ T cells from a representative experiment/donor for TNF-a plus IFN-g treatment data in Figure 5a.

Supplementary Movie 19. Videomicroscopy of $CCR6^{+(high)}CCR2^{+}$ $CD4^{+}$ T cells from a representative experiment/donor for TNF-a plus IFN-g treatment data in Figure 5a.

Supplementary Movie 20. Videomicroscopy of $CCR6^{-}CCR2^{-}$ $CD4^{+}$ T cells from a representative experiment/donor for AMG 487 control data in Figure 5b.

Supplementary Movie 21. Videomicroscopy of $CCR6^{-}CCR2^{-}$ $CD4^{+}$ T cells from a representative experiment/donor for AMG 487 treatment data in Figure 5b.

Supplementary Movie 22. Videomicroscopy of $CCR6^{+(low)}CCR2^{-}$ $CD4^{+}$ T cells from a representative experiment/donor for AMG 487 treatment data in Figure 5b.

Supplementary Movie 23. Videomicroscopy of $CCR6^{+(high)}CCR2^{+}$ $CD4^{+}$ T cells from a representative experiment/donor for AMG 487 treatment data in Figure 5b.

Supplementary Movie 24. Videomicroscopy of $CCR6^{+(high)}CCR2^{+}$ $CD4^{+}$ T cells from a representative experiment/donor for transduction with control virus in Figure 7c.

Supplementary Movie 25. Videomicroscopy of $CCR6^{+(high)}CCR2^{+}$ $CD4^{+}$ T cells from a representative experiment/donor for transduction with CCL2-CXCL9 chimera virus in Figure 7c.

Supplementary Movie 26. Videomicroscopy of $CCR6^{+(high)}CCR2^{+}$ $CD4^{+}$ T cells from a representative experiment/donor for transduction with CCL2-CXCL9 chimera virus and BMS22 control data in Figure 7c.

Supplementary Movie 27. Videomicroscopy of $CCR6^{+(high)}CCR2^{+}$ $CD4^{+}$ T cells from a representative experiment/donor for transduction with CCL2-CXCL9 chimera virus and BMS22 treatment data in Figure 7c.

Supplementary Movie 28. Videomicroscopy of $CCR6^{+(high)}CCR2^{-}$ $CD4^{+}$ T cells from a representative experiment/donor for data in Supplementary Figure 6b.

Supplementary Movie 29. Videomicroscopy of $CCR6^{+(high)}CCR2^{+}$ $CD4^{+}$ T cells from a representative experiment/donor for BMS22 control data in Supplementary Figure 6b.

Supplementary Movie 30. Videomicroscopy of $CCR6^{+(high)}CCR2^{+}$ $CD4^{+}$ T cells from a representative experiment/donor for BMS22 treatment data in Supplementary Figure 6b.

Supplementary Data 1. Antibodies used in Cellular Indexing of Transcriptomes and Epitopes by Sequencing (CITE-Seq) experiments shown in Figure 3 and Supplementary Figure 4.

Supplementary Data 2. Subject and sample data for CITE-Seq experiments shown in Figure 3 and Supplementary Figure 4.
